# Expression of miR-155, miR-146a, and miR-326 in T1D patients from Chile: relationship with autoimmunity and inflammatory markers

**DOI:** 10.20945/2359-3997000000006

**Published:** 2018-01-01

**Authors:** Diego F. García-Díaz, Carolina Pizarro, Patricia Camacho-Guillén, Ethel Codner, Néstor Soto, Francisco Pérez-Bravo

**Affiliations:** 1 Universidad de Chile Universidad de Chile Facultad de Medicina Laboratorio de Nutrigenómica, Departamento de Nutrición Chile Laboratorio de Nutrigenómica, Departamento de Nutrición, Facultad de Medicina, Universidad de Chile; 2 Universidad de Chile Universidad de Chile Facultad de Medicina Instituto de Investigaciones Materno Infantil (IDIMI), Hospital San Borja Arriarán Chile Instituto de Investigaciones Materno Infantil (IDIMI), Hospital San Borja Arriarán, Facultad de Medicina, Universidad de Chile

**Keywords:** miRNAs, inflammation, type 1 diabetes, autoimmunity

## Abstract

**Objective:**

The aim of this research was to analyze the expression profile of miR-155, miR-146a, and miR-326 in peripheral blood mononuclear cells (PBMC) of 47 patients with type 1 diabetes mellitus (T1D) and 39 control subjects, as well as the possible association with autoimmune or inflammatory markers.

**Subjects and methods:**

Expression profile of miRs by means of qPCR using TaqMan probes. Autoantibodies and inflammatory markers by ELISA. Statistical analysis using bivariate correlation.

**Results:**

The analysis of the results shows an increase in the expression of miR-155 in T1D patients in basal conditions compared to the controls (p < 0.001) and a decreased expression level of miR-326 (p < 0.01) and miR-146a (p < 0.05) compared T1D patients to the controls. miR-155 was the only miRs associated with autoinmmunity (ZnT8) and inflammatory status (vCAM).

**Conclusion:**

Our data show a possible role of miR-155 related to autoimmunity and inflammation in Chilean patients with T1D.

## INTRODUCTION

Type 1 diabetes (T1D) is an autoimmune disease triggered by T cells that destroy pancreatic beta cells. This destruction takes place by means of a complex interaction between active lymphocytes, cytokines, and macrophages (1). During the initial step of the disease, β cells are exposed to high levels of cytokines that cause the activation of the immune system and trigger the insulitis process. This inflammatory environment results in β cell damage, decreased insulin production, and the consequent destruction of β cells through apoptosis (2).

The first indication that miRNAs may be involved in regulating the β cell function was the identification of miRNAs specifically expressed in human pancreatic islets – miR-375 and miR-376 (3). In the last decade, a number of miRNAs have been described that are capable of regulating pancreatic function (4).

The expression of miRNAs may be induced by a variety of stimuli-including cell stress and inflammation, which either induce or suppress its expression in response to different stimuli, which may influence some biological processes and have pro- or anti-inflammatory effects (5) – such as hyperglycemia in patients with T1D – which increases the inflammatory response by increasing cytokines. This effect is associated with increased expression of Toll receptors (6,7), and has been correlated with studies on PBMC cultures stimulated with high glucose concentrations, which showed an increase in the expression levels of TNF-α, IL-1b, and IL-6 (8). It has been shown that stimulation by TNF-α induces the expression of certain miRNAs, including miR-146a and miR-155, which affect the pathogenesis of some diseases such as rheumatoid arthritis (6,9). Studies show the involvement of miR-155 in the activation and maturation of T and B lymphocytes. This is why it has been associated with many autoimmune diseases, such as rheumatoid arthritis; thus, an increase in the expression level of this miRNA is observed both in fibroblasts and in the PBMC of patients with this disease (10). miR-146a and miR-155 are described to be altered in T lymphocytes of patients with rheumatoid arthritis (9). miR-326 is observed to be altered in PBMC of multiple sclerosis patients (10) and shows higher expression levels in T1D patients from Italy (11,12). The aim of this study was to analyze the expression levels of the miRNAs miR-146a, miR-155, and miR-326 in PBMC from T1D and healthy patients, and to estimate their possible relationships with inflammatory or autoimmunity status in Chilean children with T1D.

## SUBJECTS AND METHODS

### Subjects

This study involves 47 T1D patients aged 6–11 years from the metropolitan region of Santiago in Chile, recruited from the Institute of Maternal and Child Research (IDIMI) of the San Borja Arriarán Hospital. T1D was diagnosed based on the American Diabetes Association (ADA) criteria. In all cases, a survey was applied to gather the patient's family medical and clinical history. The presence of possible chronic complications in T1D patients was corroborated through a survey and through the hospital clinical history; this included normal renal function (microalbuminuria) and normal eye fundus. In addition, 39 samples from healthy individuals (control group) aged 13–30 years were used. During the blood sample collection, patients and controls who declared the presence of previous febrile state (three days) or some inflammatory process were excluded from the study. The blood samples of T1D patients and controls were collected in the hospital after an informed consent was signed by parents of patients younger than 10 years and/or directly by patients older than 10 years. This study has been approved by the Ethics Committee of IDIMI and Faculty of Medicine, University of Chile.

### Extraction and culture of PBMC

The 10 ml of drawn blood was diluted with phosphate buffered saline (PBS) at a ratio of 1:1 to facilitate the handling of the sample. The PBMC was extracted and incubated, as previously described (13).

### Extraction of total RNA and miRNAs analysis

Total RNA extraction was performed using the TRIZOL method (Invitrogen) following the manufacturer's instructions. Single-stranded cDNA was synthesized from 300 ng of total RNA taken at dilutions of 2–10 ng of RNA in each sample. To assess the relative expression of miRNAs, stem-loop RT realtime PCR was performed (Applied Biosystems, Foster City, CA, USA) with specific primers for each miRNA. Expression levels were determined using TaqMan MGB probes and TaqMan Universal PCR Master Mix II (2x) in triplicate in an equipment from Agilent Technologies (CA, USA). The expression levels of miRNAs – miR-155, miR-146a, and miR326 – were normalized to a small RNA called RNU48, as an internal control.

### Serological analysis

Anti-GAD65, anti-IA2, and anti-ZnT8 antibodies were determined through enzyme immunoassay (ELISA) using the Medizym commercial kits (Berlin, Germany). Antibody detection was carried out semiquantitatively through reference to the value of 5 IU/mL for GAD65, 10 IU/mL for IA2, and 15 IU/ml for ZnT8. The analysis of inflammatory markers included human ultrasensitive C Reactive Protein (usCRP, BioVendor, Czech Republic) and the measurements of TNFα, IL-6, vCAM, and C-peptide concentrations determined by ELISA (R&D Quantikine Human ELISA Assay, UK). HbA1c levels were measured using a commercially available automatic system (DCA 2000, Bayer Diagnostics, Tarrytown, NY, USA).

### Statistical analyses

We used the REST^©^ (Relative Expression Software Tool) program, designed especially for analyzing the results of qPCR using the Pfaffl equation. Afterwards, tests were performed to evaluate the statistical significance or non-significance of the results, regarding the variations in expression observed between patients and controls. All subsequent calculations were performed using the Graph Pad Prism 6 (Graph Pad Software, Inc. San Diego CA, USA). The Shapiro-Wilk normality test was used and the effect of glucose was studied in GraphPad using the Kruskal-Wallis test. To determine the relationship between gene expression and clinical records, the bivariate correlation test was used. A p value of < 0.05 was considered as statistically significant.

## RESULTS

Table 1 describes the clinical, immunological, and inflammatory characteristics of all individuals included in this study. T1D patients showed a high pattern of autoimmunity and a pattern of 28% inflammation by mean of usCRP over 3 mg/dL. This was not observed in the control group. All controls subjects tested negative to autoantibodies profile. TNFα, usPCR, IL-6, and vCAM were significantly elevated in T1D patients compared to control subjects.

**Table 1:** Clinical, immunological and inflammatory parameters in T1D patients and controls

	T1D patients (n = 47)	Healthy controls (n = 39)	p-value
Age (years)	15.5 ± 3.9	19.5 ± 7.7	NS
BMI (kg/m^2^)	23.8 ± 3.3	25.6 ± 3.2	NS
Glycemia at debut (mmol/L)	31.3 (17.7 – 58.3)	-	-
HbA1c (%)	8.6 (6.7 – 15.5)	-	-
C-peptide (pmol/L)	94 ± 29	762 ± 314	0.01
Disease duration (years)	3.4 ± 1.9	-	-
Chronic complications*	Negative	-	-
Positive anti-ZnT8 (%)	67	Negative	-
Positive anti-GAD65 (%)	76	Negative	-
Positive anti-IA2 (%)	81	Negative	-
TNF-α (pg/mL)	4.2 ± 1.6	2.4 ± 1,3	0.01
usCRP (ng/mL)	1.71 (0.19 – 14.1)	1.28 (0.4 – 2.7)	0.03
IL-6 (pg/mL)	2.16 (0.93 – 5.61)	0.87 (0.72 – 1.44)	0.05
vCAM (ng/mL)	276.5 (101.6 – 567.9)	139.4 (91.7 – 349.2)	0.01

* Renal function (normal microalbuminuria); diabetic retinophaty: eye fundus examination.

**Figure 1:**
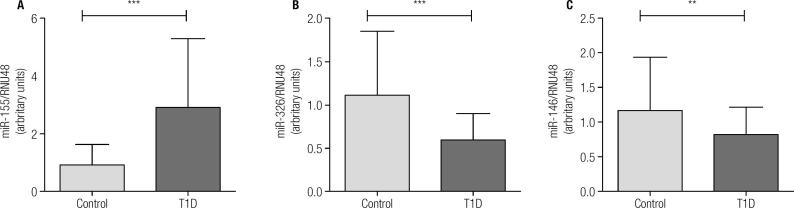
Expression of miR-146a (a); miR-55 (b) and miR-326 in control subjects (n = 37) and T1D patients (n = 47) in baseline conditions. Kruskal Wallis, Dunn post hoc test. ** p < 0.01; *** p < 0.001.

**Figure 2:**
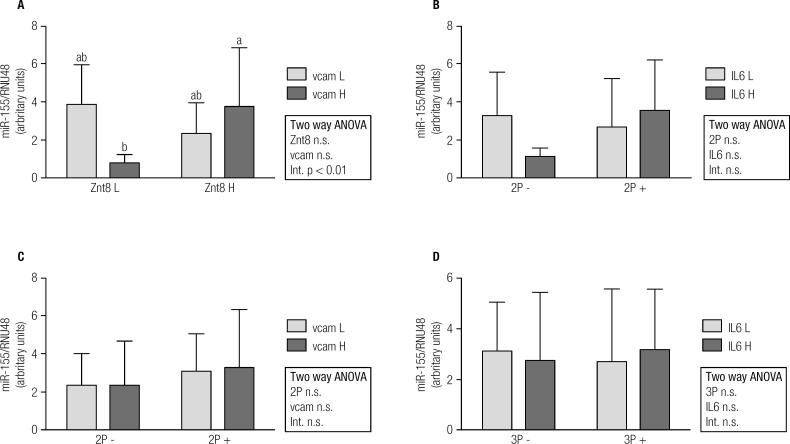
miR-155 gene expression and relationship with autoimmune and inflammatory status in T1D patients (L = low; H = high; 2P- = two negative autoantibodies; 2P+ = two positive autoantibodies; 3P- = three negative autoantibodies; 3P+ = three positive autoantibodies).

**Figure 3:**
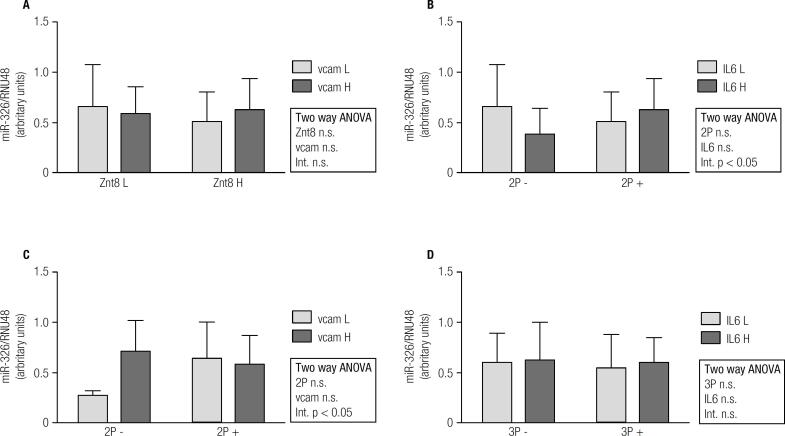
miR-326 gene expression and relationship with autoimmune and inflammatory status in T1D patients (L = low; H = high;2P- = two negative autoantibodies; 2P+ = two positive autoantibodies; 3P- = three negative autoantibodies; 3P+ = three positive autoantibodies).

**Figure 4:**
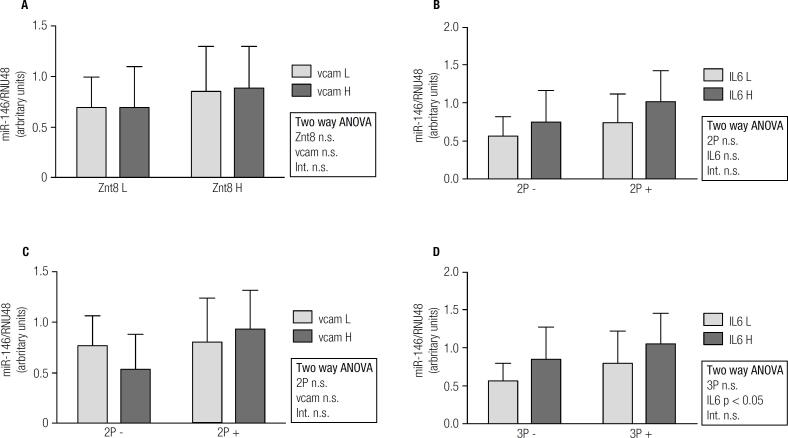
miR-146a gene expression and relationship with autoimmune and inflammatory status in T1D patients (L = low; H = high; 2P- = two negative autoantibodies; 2P+ = two positive autoantibodies; 3P- = three negative autoantibodies; 3P+ = three positive autoantibodies).

Overall, miR-155 expression was significantly higher in T1D patients than in controls (Figure 1A). On the contrary, miR-326 and miR-146a expressions were lower in T1D subjects (Figures 1B and 1C). In order to find relationships in miRNAs expression regarding autoantibody and inflammatory profile in T1D patients, 2x2 ANOVA was performed (Figures 2, 3 and 4). Regarding miR-155, only a significant interaction between Znt8 low or high titer and VCAM low or high expression was observed (p < 0.01), presenting the Znt8 H/VCAM H (the higher) and the Znt8 L/VCAM L (the lower) miR-155 expression (Figure 2A). On the other hand, miR-326 presents a significant interaction when contrasted in the presence of at least two positive autoantibodies in serum with either low/high IL6 or VCAM presence (Figures 3B and C), although these interactions seem to have come from a different pattern of expression between factors (lower expression at IL-6 H and higher at VCAM H in the two positive autoantibody conditions, although no significant differences between groups were found). Finally, miR-146a expression only showed a tendency toward a higher expression induced by higher IL-6 presence (Figures 4B and D). This is especially significant when the sample is dichotomized in presence of three positive autoantibodies (Figure 4D).

## DISCUSSION

Despite the massive expansion in miRNAs studies and extensive investigation in several diseases, the role of miRNAs in T1D has only recently been explored. T1D – an eminent autoimmune disease – suggests a possible connection between these miRNAs and the immune system components.

The relationship between miRNAs and the various components of the immune system has been addressed in different autoimmune pathologies previously. miR-155 is related with the immune response of macrophages to different types of inflammatory mediators, such as TNF-α, which can induce the expression of miR-155 in macrophages and monocytes (14,15). miR-146a is associated with innate immunity and inflammation (16). In mice, these miRNAs have shown a deficiency in accordance with cytokine production after LPS stimulation (17,18). miR-146a acts by stimulating TLR4 toll-like receptors that activate TRAF6 and IRAK1 and genes that control cytokine production, thus suggesting that miR-146a participates in regulating cytokines release (19). miR-146a expression has been described as decreased in patients with T1D and is associated with high levels of GADA (20). In our previous studies, we analyzed several miRNAs in T1D and control subjects. We obtained a different expression profile in PBMC submitted to increase concentrations of glucose (13,21). The above point opens up the possibility of searching miRNAs that are capable of sensing subtle changes in glucose profiles.

It is known that hyperglycemia increases the production of pro-inflammatory cytokines like TNF-α, IL-1β, and IL-6, which act by way of NF-kB (22). The medium in which T cells are found in T1D patients is a hyperglycemic environment leading to a sustained inflammatory state. This altered environment in which T cells are found could change the expression of some miRNAs (12,14). miR-155 is a microRNA that has been previously studied in rheumatoid arthritis, where there has been an increase in the expression levels (14), as well as in the PBMC of patients with systemic lupus erythematosus (SLE) (23). miR-155 is also a miRNA that is linked to inflammation and acts through the signaling of Toll receptors, which activate TAB 2, Ik-B and whose final objective is NF-kB, which is responsible for producing pro-inflammatory cytokines such as TNF-α and IL-1β (13). TNF-α is a potent inflammatory mediator produced by T cells; it has been linked to T1D and it is over-expressed in the inflammatory phenomena (insulitis). This inflammatory phase is characterized by an infiltrate composed mainly of CD4 and CD8 T lymphocytes β cells (24). In our study, the basal expression of miR-155 is elevated in T1D patients compared than control subjects. Our results are similar to what has been reported in viral myocarditis in myocardial cells, where the increased expression of miR-155 has been described (25). The low expression of miR-146a observed in T1D patients could be associated with an overproduction of inflammatory cytokines, as observed in studies with mice, which show that the decrease in the expression levels of miR-146a is associated with an excessive increase in proinflammatory cytokines (TNF-α and |IL-6) in response to LPS or cytokines, as in the case of sepsis and asthma (26). Our observation is consistent with the effect that occurs in vesicular stomatitis, where the low expression of miR-146a induces the production of pro-inflammatory cytokines such as TNF-α, |IL-1β, and IL-8. All these antecedents make us presume that miR-146a could regulate inflammation through a negative feedback through NF-kB to maintain a controlled immune response (15).

Regarding miR-326, in 2011, Sebastiani and cols. (12) reported a high positive correlation between this miRNA and autoimmunity. However, this analysis was carried out only for a group of T1D patients, without comparing their miRNA levels with that of healthy subjects, as was done in the work of Du and cols. (27) on multiple sclerosis. Our study shows differences between miR-326 expression between T1D patients and controls, and no relationship based on the number of positive autoantibodies. Finally, our study shows a tendency for possible relationships between the expression of miR-22 and ZnT8 antibodies among a group of patients who tested positive for this autoantibody, which could indicate an association effect. A recent study reports that 32 miRNAs located in the same genomic region (Chromosome 14q32) could act on the mRNA of several T1D autoantigens; 12 of these miRNAs were sensitive to changes in glucose. This study shows no data on ZnT8 (28). The relationship between miRNAs and the environmental factors (virus, diet) – related with the T1D and the immune system regulation – is a field of research that is currently being explored (29).

Our study describes an increase in the expression of miR-155 and a decrease in the expression of miR-146a and miR-326 in T1D patients, compared to control subjects. A possible interaction was observed between miR-155 and ZnT8 autoantibody, but no interaction was described to inflammatory status in T1D (related with vCAM and IL-6 levels). Regarding our cell model, PBMC represent a diverse population of cells; as such, each distinct cell type may have a unique miRNA expression profile. Finally, an important aspect to be considered in our study is the age of the disease among patients with T1D. This study includes young patients with a short evolution of their disease. In general, during this period, the patients have a metabolically stable picture. Our findings should be interpreted with caution if we consider advanced stages of the disease. Several microRNAs have been linked to complications of T1D. There is evidence of changes in proinflammatory cytokines and oxidative stress in these patients, consistent with changes in glycemic stability and with changes in the miRNAs profile in according with long-standing hyperglycemia (30,31). This would be an interesting point for corroboration in future studies, with cell subpopulations to establish the true benefits and limitations of circulating miRNA as biomarkers of T1D.
